# ℤ‐Classified Topological Phases and Bound States in the Continuum Induced by Multiple Orbitals

**DOI:** 10.1002/advs.202409574

**Published:** 2025-01-21

**Authors:** Shi‐Feng Li, Wen‐Jie Yang, Cui‐Yu‐Yang Zhou, Yi‐Fan Zhu, Xin‐Ye Zou, Jian‐Chun Cheng, Badreddine Assouar

**Affiliations:** ^1^ Key Laboratory of Modern Acoustics MOE Institute of Acoustics Department of Physics Collaborative Innovation Center of Advanced Microstructures Nanjing University Nanjing 210093 P. R. China; ^2^ Jiangsu Key Laboratory for Design and Manufacture of Micro‐Nano Biomedical Instruments School of Mechanical Engineering Southeast University Nanjing 211189 China; ^3^ Université de Lorraine CNRS Institut Jean Lamour Nancy 54000 France

**Keywords:** bound states in the continuum, hybrid topological insulator, multipole chiral numbers, orbital degree of freedom, ℤ‐classified topological insulators

## Abstract

**ℤ‐**classified higher‐order topological insulators (HOTIs) with chiral‐symmetric higher‐order topological phases protected by multipole chiral numbers (MCNs) have attracted extensive interest recently. However, how to design artificial ℤ‐classified HOTIs with multiple topological phases remains an unresolved issue. Here, multiorbital degrees of freedom are introduced to acoustic crystals and the various methods of topological phase transitions are achieved for the orbital ℤ‐classified HOTIs. Experimental results demonstrate the realization the coexistence of corner modes with distinct mechanisms within one single model. This provides a pathway for finding ℤ‐classified with large MCNs independent of long‐range coupling. Additionally, a universal approach is introduced here to fabricate topological bound states in the continuum derived from the discrepant onsite energy of degenerate *p*‐orbitals. These findings provide new insights into the study of topological wave physics using orbital degrees of freedom and may pave the way for designing innovative orbital topological devices for sensing and computing.

## Graphical abstract

Various design and control methods are presented here for ℤ‐classification topological insulators using acoustic orbital degrees of freedom. Experiments here show that degenerate multiorbitals can be used to flexibly control topological phase transitions and create topological bound states in the continuum. This holds promise for the development of new acoustic devices, such as sensors.

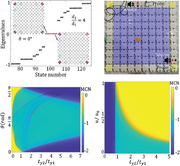

## 1 Introduction

In condensed matter systems, one important feature of electrons is the intrinsic orbital degree of freedom, which plays a crucial role in understanding the unconventional properties of solid‐state materials and in revealing the science and developing technology related to electrons.^[^ ^1^ ^]^ It gives rise to a multitude of intriguing phenomena, including metal–insulator transition,^[^ ^2^ ^]^ superconductivity,^[^ ^3^ ^]^ and colossal magnetoresistance.^[^ ^4^ ^]^ In recent years, with the increase in the research on topological states of matter, researchers have found that the orbital degree of freedom also plays a crucial role in revealing novel topological phases, such as orbital superfluidity^[^ ^5^ ^]^ and topological semimetals.^[^ ^6^ ^]^ Recently, the exploration of orbitals in topological matter has expanded into the fields of photonics,^[^ ^7^, ^8^, ^9^, ^10^, ^11^, ^12^, ^13^ ^]^ electronics,^[^ ^14^, ^15^ ^]^ and acoustics.^[^ ^16^, ^17^, ^18^ ^]^ For instance, in the field of photonics, topological lasing has been achieved in a zigzag array through edge modes supported by polariton micropillars.^[^ ^9^ ^]^ Additionally, recent experiments have shown that the *p*‐orbitals can be carefully tailored and manipulated within artificial electronic lattices created atom by atom using a scanning tunnelling microscope,^[^ ^4^, ^5^, ^6^, ^7^, ^8^, ^9^, ^10^, ^11^, ^12^, ^13^, ^14^, ^15^, ^19^ ^]^ through which a spin–orbit coupling analogy has been obtained and the *p*‐orbital bandgap flat band, as well as the Dirac cone, has been confirmed. In terms of acoustic systems, the orbitals are often used as a tool for designing artificial topological insulators to study the topological transport properties, such as the acoustic quantum spin Hall effect.^[^ ^20^, ^21^, ^22^ ^]^

Recently, an increasing number of researchers have been interested in orbital higher‐order topological insulators (HOTIs), i.e., HOTIs with multiple orbitals^[^ ^12^, ^13^, ^16^ ^]^ and realized multiorbital induced zero‐energy modes in several HOTIs.^[^ ^23^, ^24^, ^25^, ^26^, ^27^, ^28^ ^]^ All these works focus on exploring the interaction between multiorbitals and some well‐known HOTI models, such as the breathing Kagome lattice model,^[^ ^23^, ^24^ ^]^ the 2D Su‐Schrieffer‐Heeger (SSH) model^[^ ^25^, ^26^ ^]^ and so on.^[^ ^27^, ^28^ ^]^ Meanwhile, whether new artificial HOTI models can be designed using degenerate orbitals remains an open question, which could have significant implications for topological classification.

A very recent paper demonstrated the existence of a ℤ classification for HOTIs in class AIII and identified that *N* zero‐energy states at each corner are protected by multipole chiral numbers (MCNs).^[^ ^29^ ^]^ The MCNs generalize the real‐space representation of the 1D winding number to higher‐dimensional systems. In contrast to the 1D winding number, the MCNs are constructed using sublattice multipole moment operations and applicable to the boundary‐obstructed topology phases. The presence of MCN‐protected phases enhances the understanding of the HOT phases beyond the Wannier center and mass domain perspectives^[^ ^29^, ^30^, ^31^, ^32^, ^33^, ^34^ ^]^ and has attracted considerable interest.^[^ ^35^, ^36^, ^37^, ^38^, ^39^, ^40^, ^41^ ^]^ However, HOTIs with MCNs greater than 1 require strong long‐range hopping,^[^ ^29^, ^35^, ^36^, ^37^ ^]^ which increases the complexity of device design and practical applications of HOTIs. The following question thus naturally arises: can we realize ℤ‐classified HOTIs without reliance on long‐range hopping by multiple orbitals, and how to conveniently control the topological phase transitions to suit the design and applications needs?

In this work, we theoretically and numerically calculate the eigenmodes of the *p*‐orbital 2D SSH acoustic lattice, revealing the topological phase transitions induced by multiple orbitals. Furthermore, using orthogonal *p*‐orbitals, we construct an acoustic crystal with the combined topological properties of both the 2D SSH model and the quadrupole insulator, referred to as the hybrid topological insulator (HTI). Upon adjustment of the π‐bonding strength, the number of corner states in each corner is expected to vary, which demonstrates the variation in the MCNs. We thus realize several designs of multiple‐orbital‐induced ℤ‐classified HOTIs and achieve MCNs > 1 without relying on long‐range coupling. Besides, the degenerate orbital acoustic lattices provide a platform for the investigation of orbital‐dependent topological bound states in the continuum (TBICs). The orthogonal nature of the degenerate *p*‐orbitals leads to the possibility of constructing TBICs and has been confirmed in our experiment. It is worth noting that, unlike previous reports on multiorbital topological insulators, our work focuses on designing novel artificial ℤ‐classified HOTIs and exploring feasible methods for manipulating their phase transitions. This not only provides insights for realizing topological phases beyond the tenfold classification but also paves the way for designing easily operable orbital acoustic devices, such as high‐sensitivity sensors.^[^ ^42^, ^43^, ^44^ ^]^

## 2 Orbital 2D SSH Model

**Figure 1:**
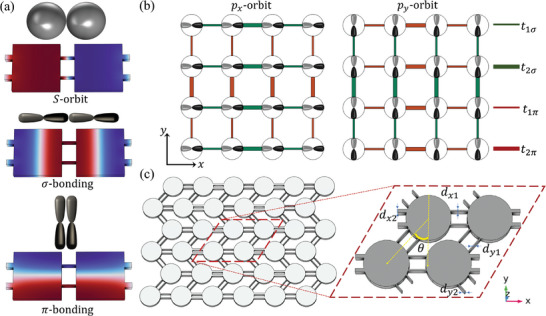
a) Schematics and spatial field distributions of the *s*‐orbital and two degenerate *p*‐orbital eigenmodes for the dimer cavities. Multiple *p*‐orbitals lead to two different coupling strengths: σ bonding and π bonding. b) Schematic diagram of *p*‐orbital 2D SSH model. The thin and thick lines represent the intra‐ and intercell couplings, respectively, and the green and red lines represent the σ and π bonding, respectively. c) Schematic diagram of 2D SSH acoustic crystal. The enlarged inset shows the unit cell of the acoustic crystal when θ  =  45°.

**Figure 2:**
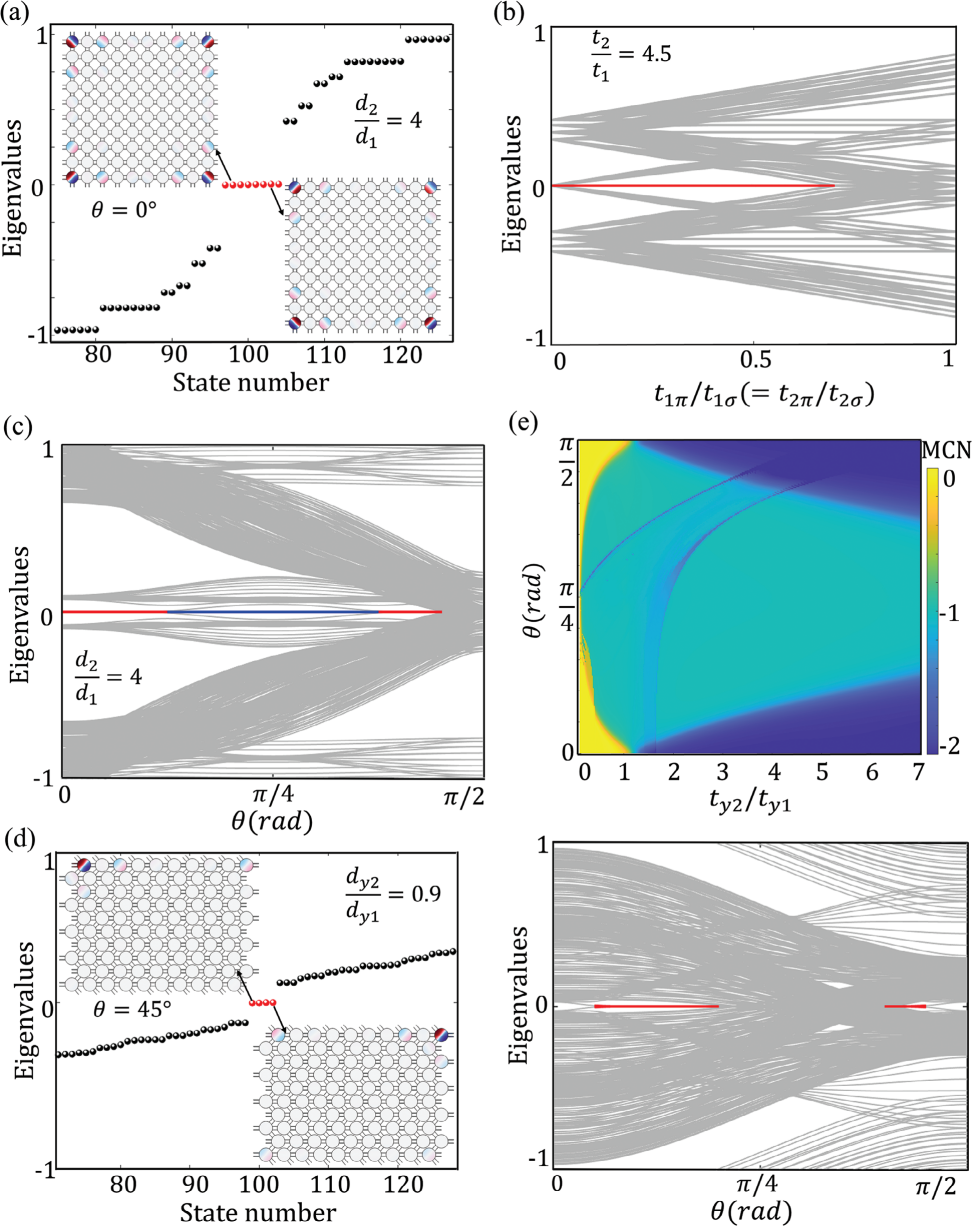
a) Normalized eigenvalues of the orbital 2D SSH model with θ  =  0. The insets show the spatial distribution for two of the eight corner states obtained from numerical calculations. b) Eigenvalue spectrum as a function of *t*_π_/*t*_σ_. c) Eigenvalue spectrum as a function of θ with *d*_2_/*d*_1_ = 4. The red and blue lines mark the zero energy modes of eightfold and fourfold degeneracy, respectively. d) Eigenvalues of the acoustic crystal with θ  =  45° and *d*_*y*2_/*d*_*y*1_ = 0.9 and eigenvalue spectrum as a function of θ. The insets show the spatial distribution of corner states. e) Topological phase diagram of the 2D SSH model.

In this work, we consistently use subscripts 1/2 to represent intra/intercell coupling, *x*/*y* to represent directions, and σ/π to represent the two modes of *p*‐orbital coupling. To determine whether novel topological effects can be achieved, we focus on acoustic HOTIs by establishing their *p*‐orbital models. Similar to the discrete *s* and *p*‐orbital energy levels of hydrogen atoms, acoustic cavities with *C*_4_ symmetry support not only the zero‐mode but also degenerate *p*‐orbital resonances, which are associated with two types of dipole modes, *p_x_* and *p_y_* modes. Multiple *p*‐orbitals lead to two different coupling strengths between resonators: those for σ bonding and π bonding,^[^ ^16^, ^17^, ^18^ ^]^ as shown in **Figure** **1**a,b. Here we begin with the well‐known 2D SSH model, whose unit cell of the acoustic crystal consists of four sublattices, as depicted in Figure 1c. The intra‐ and intercell hopping amplitudes, *t*_1_ and *t*_2_, are controlled by the widths of the coupling tubes denoted as *d*_*x*1_, *d*_*x*2_, *d*_*y*1_, and *d*_*y*2_. The angle between the *y*‐axis and the bonding along the *y*‐direction, i.e., θ is conventionally set to 0, as it has no impact on the topological properties of the single‐orbital model. Based on tight‐binding model theory, we obtain the Hamiltonian (see additional details in the Supporting Information) of this acoustic crystal and calculate the normalized eigenenergy spectrum for *d*_*x*1_ = *d*_*y*1_ = 1 and *d*_*x*2_ = *d*_*y*2_ = 4, as shown in **Figure** **2**a (only 50 eigenvalues are shown here for clarity). In the conventional single‐orbital 2D SSH model respecting *C*_4_ symmetry (see additional details in the Supporting Information), due to isotropic hopping *t_x_* = *t_y_*, the bulk bandgap will close and obscure the zero‐energy modes. But this is avoided in the *p*‐orbital model because the difference between σ‐bonding and π‐bonding breaks the isotropy as shown in Figure 1b. Thus, eight red dots in Figure 2a marking the degenerate orbital corner modes appear in the bandgap of the eigenenergy spectrum, although the entire model respects *C*_4_ symmetry. The simulation results (insets of Figure 2a) show that the corner modes are exponentially localized at the sublattice sites with an opposite phase between the dipoles in the corner and the two next‐nearest‐neighbor sites, which is a characteristic feature of HOTI corner modes. The eigenvalue spectrum as a function of the ratio of σ‐bonding to π‐bonding, i.e., *t*_π_/*t*_σ_, is shown in Figure 2b. It can be seen that as the strengths of these two types of bonding get approaching, the bulk energy bandgap gradually shrinks until it closes, and the zero‐energy modes marked by the red line are screened. This is foreseeable because when *t*_π_ equals *t*_σ_, the system resembles two uncoupled *s*‐orbital 2D SSH models, implying the preservation of isotropy.

More interesting thing happens when θ deviates from 0 and the unit cell becomes a rhombus, leading to zigzag boundaries at the ends in the *x*‐direction. The number of topological corner states varies with θ, as shown in the right panel of Figure 2c, where the eightfold degenerate zero‐energy modes (red line) reduce to fourfold degenerate modes (blue line) in the interval (π/8,  3π/8). The variation in the number of topological corner states implies a topological phase transition, although the condition *d*_2_/*d*_1_ = 4 that symbolizes nontrivial topology in the single‐orbital model remains unchanged. In fact, the variation in θ not only induces a decrease in the number of topological corner states in the nontrivial 2D SSH model but also leads to the generation of corner states in the topologically trivial model. As shown in Figure 2d, when *d*_2*y*_ = *d*_1y_*0.9, meaning that the system is topologically trivial, the fourfold degenerate zero modes marked by the red line appear in the interval (0.06π,  0.44π), although in some regions they may be obscured by closed bulk gaps. The left panel of Figure 2d shows that the fourfold degenerate topological corner states appear at both acute and obtuse corners when θ  =  45°, and the dipole distribution of the acoustic pressure inside the cavities confirms the π‐bonding in the *y*‐direction.

To further theoretically demonstrate the topological phase transition induced by the *p*‐orbital, we calculate the topologically invariant MCNs. For 2D chiral‐symmetric systems, MCNs are defined as

(1) $$N_{xy}=\frac{1}{2\pi i}\mathrm{Tr}\log\left({\bar{Q}}_{xy}^{A}{\bar{Q}}_{xy}^{B^{†}}\right)$$

where ${\bar{Q}}_{xy}^{S}=U_{s}^{†}Q_{xy}^{S}U_{S}$ is the sublattice multipole moment operator $Q_{xy}^{S}$ projected into the space *U_S_* of the sublattice *S*  =  *A*,  *B*. For a 2D lattice of *L_X_* × *L_Y_* unit‐cells, $Q_{xy}^{S}=\sum_{R,\alpha \in S}\left|R,\alpha \rangle \exp\left(-\frac{i2\pi xy}{L_{X}L_{Y}}\right)\langle R,\alpha \right|$, with *R*  =  (*x*, *y*) labeling the unit‐cell of the finite lattice. Figure 2e presents the phase diagram characterized by MCNs, which is plotted with the inter/intracell hopping ratio *t*_*y*2_/*t*_*y*1_ and the angle θ (10*10 cells are taken to calculate the MCNs). This phase diagram clearly demonstrates the orbital‐induced ℤ‐classified HOTI, as θ is unrelated to the topological phase transitions in the conventional single‐orbital 2D SSH model. The two noninteger MCN bands appearing in the phase diagram are caused by closure of the bulk bandgap, i.e., the system is in a metallic phase under these parameters (which is not relevant to what is discussed in this work, additional details in the Supporting Information). These properties of orbital HOTIs can provide insights for the design of novel devices that allow easier topological control.

## 3 Demonstration of Large‐MCN in HTI

**Figure 3:**
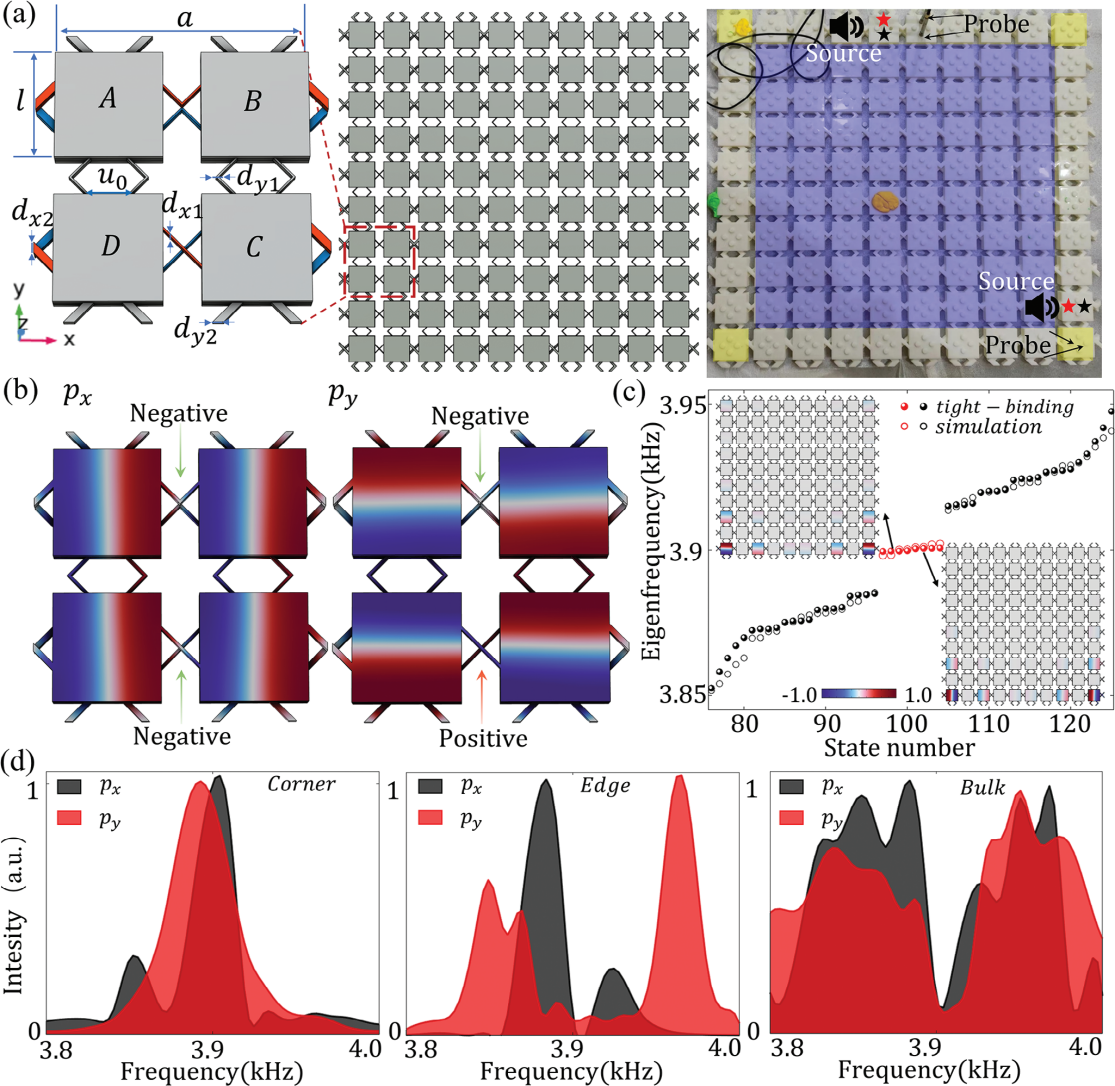
a) Designed HTI acoustic crystal and its unit cell. The right panel shows the sample used in the experiment, with cavities located at the corners, edges, and bulk marked in yellow, white, and blue, respectively. Colored stars mark a pair of out‐of‐phase speakers as the dipolar source and the corresponding two detectors are used to measure the sound responses. b) For the *p_y_* mode, positive coupling occurs between the top two cavities in the unit cell, whereas negative coupling occurs between the bottom two cavities. For the *p_x_* mode, the two couplings are both negative. c) The dots and circles, respectively, mark the eigenvalues for the HTI obtained by tight‐binding model theoretical and numerical calculations. The inset displays the field distributions of two corner states, with one being the *p_x_* mode and the other *p_y_*. d) Signals are stimulated by successively using *p_x_* and *p_y_* mode sources and the corresponding responses in the cavities located at the corner, edge, and bulk are measured.

Next, we demonstrate another kind of orbital‐induced ℤ‐classified chiral‐symmetric HOTI, called an HTI, that combines the properties of both the 2D SSH and the quadrupole insulator and lacks any single‐orbital counterpart as a result. As shown in **Figure** **3**a, the unit cell of the acoustic HTI crystal consists of four identical cavities (labeled *A*,  *B*,  *C*, and *D*) with a size of *l*  =  22 mm, a lattice constant of *a*  =  120 mm, and intra‐ and intercell coupling tube widths along the *x* and *y* directions given by *d*_*x*1_ = *d*_*y*1_ = 0.77 mm, and *d*_*x*2_ = *d*_*y*2_ = 3.08 mm, respectively. The sublattices *A* and *D* are coupled by two V‐shaped tubes folded at 90° and the same arrangement applies to *B* and *C* as well as to *A* and *B*. In contrast, between *C* and *D*, there are two straight pipes perpendicular to each other that are staggered vertically. This difference leads to a result: for the *y*‐polarization labeled by *p_y_*, the couplings between *A* and *B* and between *C* and *D* are positive and negative,^[^ ^18^, ^45^ ^]^ respectively, while for the *x*‐polarization labeled by *p_x_*, both couplings are negative, as shown in Figure 3b. All coupling tubes have the same length and spacing *u*_0_ = 7.78 mm, which determine the π‐bonding strength. The intercell tubes are the same as the intracell tubes except for the width, which, as mentioned earlier, is four times that of the intracell tubes. The Hamiltonian for this acoustic HTI crystal based on the tight‐binding model reads as

(2)

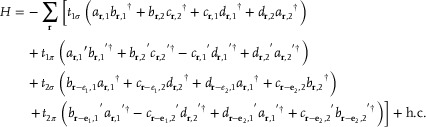

here, $a_{r}/{a_{r}}^{\prime }$, $b_{r}/{b_{r}}^{\prime }$, $c_{r}/{c_{r}}^{\prime },$ and $d_{r}/{d_{r}}^{\prime }$ are the σ/π types of projection operators associated with sites *A*,  *B*,  *C*, and *D* in a unit‐cell, respectively, located at position ***r***. The subscript *i* in operator *a*_*r*,*i*_ denotes the direction of the projection along the primitive lattice vector *e_i_*, with *i*  =  1,  2. *t*_1σ_ and *t*_1π_ (*t*_2σ_ and *t*_2π_) are the hopping amplitudes for the intracell‐ (intercell‐) σ and π types, which are determined by the widths of the intracoupling‐ (intercoupling‐) tubes *d*_*x*1_ and *d*_*y*1_ (*d*_*x*2_ and *d*_*y*2_), un in Figure 3a. The Hamiltonian demonstrates that the acoustic crystal has chiral symmetry. The simulated eigenfrequencies of the HTI are shown in Figure 3c, with the eight topological corner states marked by red dots, half of which are *p_y_* modes corresponding to the four zero‐energy modes of the quadrupole insulator, while the others are *p_x_* modes corresponding to those of the 2D SSH model. The simulation results are in good agreement with the tight‐binding theory results. We provide the Hamiltonian in momentum space in the Supporting Information, which clearly illustrates its combination of the topological quadrupole insulator and the 2D SSH model.

The emergence of topologically protected orbital‐induced corner states in the acoustic lattice was validated in experiments. We used two sets of orthogonal orbital sources^[^ ^16^, ^17^, ^18^ ^]^ to excite the acoustic field (see experimental details in the Supporting Information), with each set consisting of two out‐of‐phase sound signals. As shown in the left panel of Figure 3d, the *p_y_* and *p_x_* modes measured at the corners reach their peak responses at 3890 and 3905 Hz, respectively. The slight deviation primarily stems from 3D printing errors in the sample and the small differences in the acoustic coupling between the V‐shaped and straight tubes. Here, the combination of the topological quadrupole insulator and the 2D SSH model is achieved, showing for the first time the corner states of different mechanisms on a single HOTI, which implicates a new topological classification. Additionally, the experiment undoubtedly demonstrates that even in the absence of long‐range hopping,^[^ ^30^, ^34^, ^35^ ^]^ HOTIs can exhibit MCNs greater than 1, which is symbolized by multiple degenerate topological corner states at each corner. Our work can also be extended to 1D topological insulators to realize large winding numbers without the need for long‐range hopping.^[^ ^46^ ^]^

## 4 Visualization of Topological Phase Transitions in HTI

**Figure 4:**
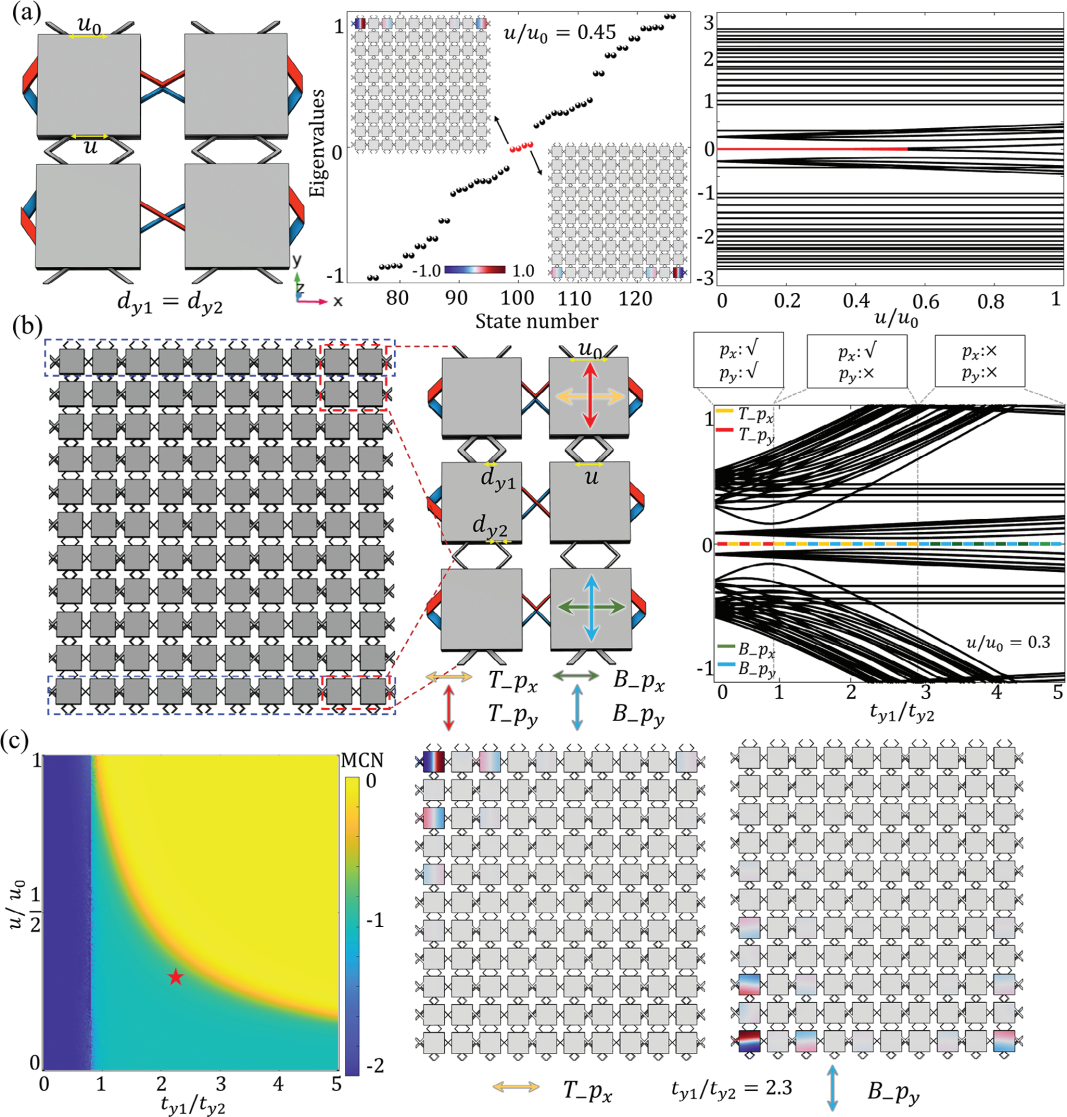
By varying the spacing *u* between the coupling tubes, the ratio of the σ‐ and π‐bonding strengths could be adjusted. a) Simulated eigenvalues of the HTI when *u*/*u*_0_ = 0.45, and eigenvalue spectrum as a function of *u*/*u*_0_. The zero modes are marked in red. The left panel presents a unit‐cell with varying *u*. b) Sketch of the 5*6 HTI with the cavities in the bottom row cropped. The top corner states of the *p_x_* and *p_y_* modes are marked in yellow and red, respectively, while the green and blue colors mark those at the bottom. The right panel displays the energy spectrum as it varies with *t*_*y*1_/*t*_*y*2_. The tick and cross respectively represent topologically nontrivial and trivial. The corner states of the *p_x_* and *p_y_* modes undergo tunneling separately as a result of the topological phase transition. c) Phase diagram obtained by calculating the MCNs as a function of *t*_*y*1_/*t*_*y*2_ and *u*/*u*_0_. The corner states of the *p_x_* and *p_y_* modes with *t*_*y*1_/*t*_*y*2_ = 2.3 (*d*_*y*1_/*d*_*y*2_ = 3) and *u*/*u*_0_ = 3 are shown in the middle and right panels, respectively.

In the previous section, we reported that the phase transition of orbital HOTIs can be induced by adjusting the width of the coupling tubes and the angle θ of the *p*‐orbital bond. Here, we introduce another method, which involves adjusting the ratio of the π‐bonding and σ‐bonding strengths by varying the spacing *u* between the coupling tubes. For the previously mentioned HTI, when the intercell hopping equals the intracell hopping, it is topologically trivial, i.e., characterized by the lack of zero‐energy corner states. However, by adjusting the spacing of the intracell coupling tubes *u* (only the *y*‐direction is considered here for simplicity as shown in **Figure** **4**a, discussions for the *x*‐direction can be found in the Supporting Information), fourfold degenerate corner states can be induced in the bandgap. The corner states marked by the red dots in the middle panel of Figure 4a exhibit *p_x_* modes, demonstrating the correspondence to the zero‐energy modes of the 2D SSH model. Additionally, in the right panel, the red line marking the zero‐energy modes gradually merges into the edge modes beyond approximately *u*/ *u*_0_ = 0.5, indicating that these corner modes are generated by the topological phase transition of the edge modes.^[^ ^47^ ^]^

When we further adjust the width of the coupling tubes *d_y_* to vary the hopping amplitude *t_y_*, a more interesting phenomenon never found in single‐orbit HOTIs will happen: the orbital corner states tunnel through the acoustic crystal, demonstrating the visual topological phase transition of the HTI. As shown in Figure 4b, the bottom row of cavities is removed from a 6*5 size HTI, leading to the top and bottom rows of cavities (including their connected coupling tubes), which are enclosed by the blue dashed boxes, in the remaining acoustic crystal being identical. The *p_x_* and *p_y_* mode corner states localized at the top corners are marked in yellow and red, respectively, and similarly, the corner states of the *p_x_* and *p_y_* modes at the bottom are marked in green and blue, respectively. When we keep the distance between the intracell tubes constant at *u*  =  0.3* *u*_0_ and vary their widths, the distribution of the topological corner states changes twice, signifying two topological phase transitions, as shown in the right panel of Figure 4b. The first phase transition occurs at approximately *t*_*y*1_/*t*_*y*2_ = 0.95, beyond which the *p_y_* mode corner states tunnel from the top corners to the bottom, while the *p_x_* mode corner states remain unchanged at the top. The second phase transition occurs at approximately *t*_*y*1_/*t*_*y*2_ = 2.9, after which the *p_x_* mode corner states will also tunnel to the bottom.

To theoretically demonstrate these phase transitions, we calculate the MCNs and plot the phase diagram with respect to *u* and *t*_*y*1_/*t*_*y*2_, as shown in Figure 4c. The twice color transitions on the phase diagram at *u*  =  0.3*u_0_ correspond well to twice corner‐state tunnelling events aforementioned. We also numerically calculate the HTI eigenmodes at the parameter point marked by the red star, i.e., (*t*_*y*1_/ *t*_*y*2_, *u*/*u*_0_)  =  (2.3,  0.3), as shown in the middle and right panels of Figure 4c, where the *p_x_* mode corner states are localized at the top corners, corresponding to the zero modes of the 2D SSH model, and the *p_y_* mode corner states at the bottom correspond to the quadrupole insulator. These abundant topological phase transitions and diverse means of topological phase manipulation, which are not available in conventional HOTIs, demonstrate the promising application of orbital HOTIs in the development of novel topological devices.

## 5 Bound States in the Continuum

**Figure 5:**
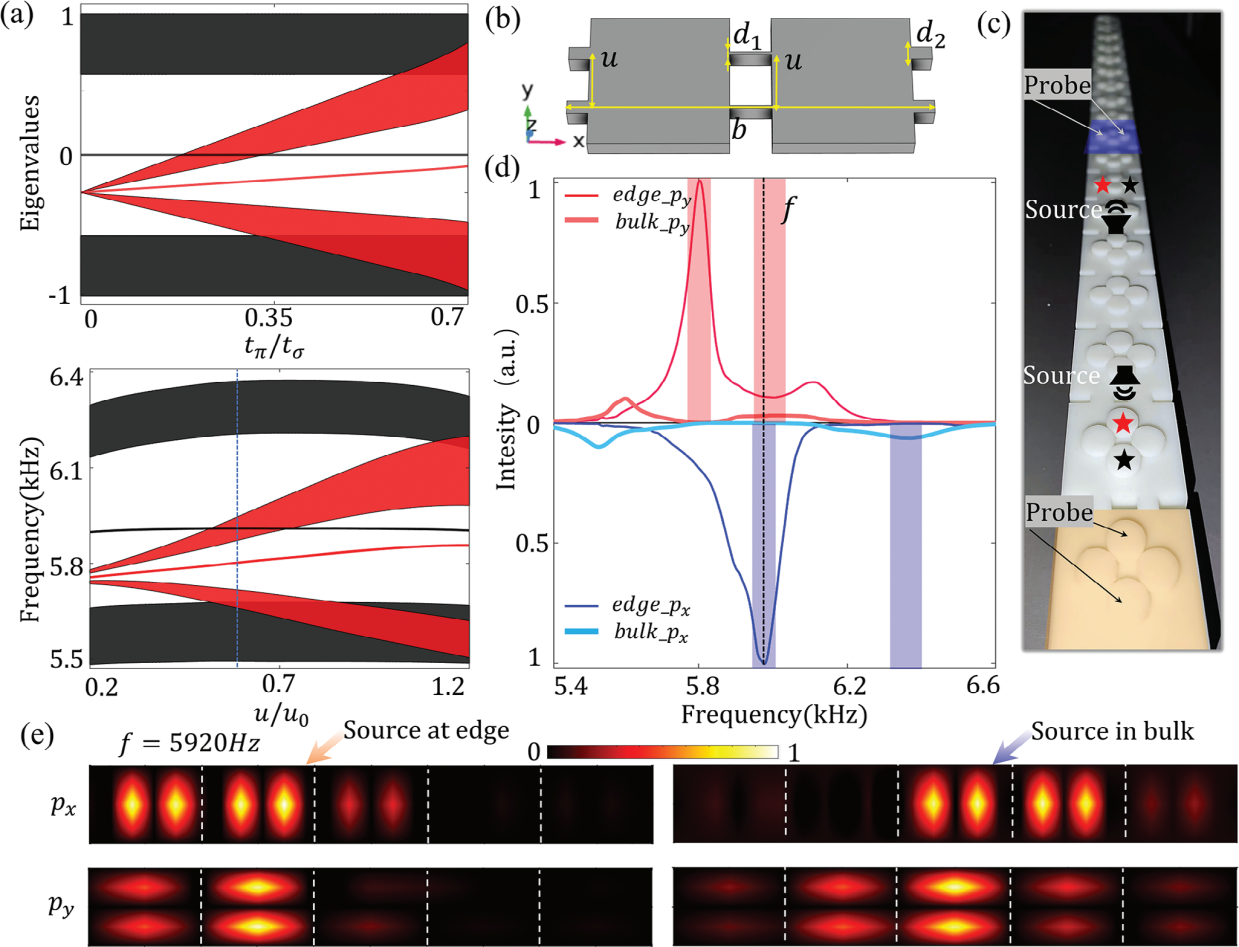
a) Eigenfrequency spectrums of the SSH chain calculated with the tight‐binding model theory and simulation. Black and red marks the *p_x_* and *p_y_* modes, respectively. b) Unit‐cell of the orbital SSH chain. c) The experimental sample of the orbital SSH model, with yellow and blue marking a cavity on the edge and bulk, respectively. Colored stars mark the dipolar source and two detectors are used to measure the sound responses. d) Average intensity spectra measured for the bulk and edge states with different excitations. The dashed line highlights the TBIC frequencies *f*. The measured quality factor is *Q*  =  38.82. e) Measured intensity patterns at *f* with different excitations. Only five cavities on the edge and middle are displayed, respectively.

BICs as spatially localized states with energies lying in the continuum of extended modes have been widely investigated in both quantum and classical systems.^[^ ^48^, ^49^, ^50^ ^]^ Recently, a universal method for constructing TBICs using layer degrees of freedom (DoFs) has been proposed.^[^ ^35^ ^]^ Similarly, orbital DoFs also provide a good platform for TBICs, and meanwhile avoid the complex structures brought by double‐layer stacking, which makes it difficult for practical applications. We take the orbital 1D SSH chain consisting of *N* unit‐cells as an example to introduce the principle of orbital DoFs inducing topological TBICs, whose real‐space Hamiltonian is

(3) $$H=\left[\begin{array}{cc} D_{\sigma } & 0\\ 0 & D_{\pi } \end{array}\right]$$

where

(4) $$D_{\sigma }=\left[\begin{array}{ccccccc} T_{1} & t_{1\sigma } & 0 & 0 & \cdots & 0 & 0\\ t_{1\sigma } & T_{2} & t_{2\sigma } & 0 & \cdots & 0 & 0\\ 0 & t_{2\sigma } & T_{3} & t_{1\sigma } & \cdots & 0 & 0\\ 0 & 0 & t_{1\sigma } & T_{4} & \cdots & 0 & 0\\ \cdots & \cdots & \cdots & \cdots & \cdots & t_{1\sigma } & 0\\ 0 & 0 & 0 & 0 & t_{2\sigma } & T_{N-1} & t_{2\sigma }\\ 0 & 0 & 0 & 0 & 0 & t_{1\sigma } & T_{N} \end{array}\right]$$

and

(5) $$D_{\pi }=\left[\begin{array}{ccccccc} T_{1}^{\prime } & t_{1\pi } & 0 & 0 & \cdots & 0 & 0\\ t_{1\pi } & T_{2}^{\prime } & t_{2\pi } & 0 & \cdots & 0 & 0\\ 0 & t_{2\pi } & T_{3}^{\prime } & t_{1\pi } & \cdots & 0 & 0\\ 0 & 0 & t_{1\pi } & T_{4}^{\prime } & \cdots & 0 & 0\\ \cdots & \cdots & \cdots & \cdots & \cdots & t_{1\pi } & 0\\ 0 & 0 & 0 & 0 & t_{2\pi } & T_{N-1}^{\prime } & t_{2\pi }\\ 0 & 0 & 0 & 0 & 0 & t_{1\pi } & T_{N}^{\prime } \end{array}\right]$$

are two decoupled sub‐matrices of size N*N, pertaining to the *p_x_* and *p_y_* modes, respectively. In the Hamiltonian matrix, *t*_1/2σ_ and *t*_1/2π_ respectively represent the strength of the σ‐bonding and π‐bonding intra/inter unit‐cells, *T* and *T*′ as each onsite‐potential of these two sub‐matrices are proportional to the sum of squares of their neighboring matrix elements.^[^ ^51^ ^]^ The fact that *t*_σ_ is usually inequal to *t*_π_, results in the difference between the onsite‐potential *T* and *T*′. This means that although they each obey chiral symmetry, the “zero‐energy” modes the two sub‐matrices *D*_σ_ and *D*_π_ are not identical. As shown in **Figure** **5**a, as *t*_π_ gradually approaches *t*_σ_, the frequencies of the topological edge states marked by the black line for the *p_x_* mode and the red line for the *p_y_* mode also gradually converge, during which, the topological edge states of the *p_x_* mode continuously pass through the continuum of the *p_y_* mode inevitably, indicating the generation of a TBIC. We numerically calculate the eigenfrequencies of the orbital acoustic crystal with unit‐cells as shown in Figure 5b and the result shown in the lower panel of Figure 5a is consistent with the tight‐binding model calculation result.

In the experiment, the acoustic crystal we constructed, as shown in Figure 5c, has the following parameters: the width of the intracell coupling tubes is *b*_1_  =  0.75 mm, the intercell is *b*_2_ =  3 mm, the lattice constant is *b*  =  78 mm, and the distance of coupling tubes is *u*  =  6.45 mm. We input acoustic signals of the *p_x_* and *p_y_* modes separately into the cavities inside the unit‐cell at the boundary and the center, then scan the frequencies and measure the responses. As shown in Figure 5d, the acoustic response measured inside the edge cavity excited by the *p_x_* mode source, is characterized by the thin blue line and reach its peak at the frequency of *f*  =  5920 Hz. Meanwhile, this frequency lies within the continuum bulk response of the *p_y_* mode characterized by the thick red line, which demonstrates the existence of the TBIC. We also measured the acoustic pressure field distribution at 5920 Hz. As shown in Figure 5e, the left two panels display the distribution in the five outermost cavities excited by the *p_x_* and *p_y_* mode sources, respectively, while the right two panels display that in the five cavities in middle. When the *p_x_* mode source is placed in the second cavity at the edge, the acoustic energy is prominently localized in the cavities on its left and right sides, proving that this frequency is precisely the edge state frequency. Similarly, when the *p_y_* mode source is placed in the cavity of the bulk unit‐cell, the acoustic energy will obviously spread on both sides, proving that this frequency is also the bulk state frequency. It is important to emphasize that this method of constructing TBICs is not only applicable to 1D TIs but also HOTIs as long as the π‐bonding can cause the different shift of zero‐energy modes from the σ‐bonding, such as Kagome lattice (more information in the Supporting Information). And our method for creating TBICs can be readily expanded to various wave systems, such as photonics, elastic‐mechanical systems, and so on.

## 6 Conclusion

In this work, we show that ℤ‐classified topological phases can be induced by *p*‐orbitals. In the orbital 2D SSH model, topological phase transitions occur when both the hopping ratio and the bond angle are tuned, which is different from the conventional single‐orbital model. With the assistance of the carefully designed HTI, we observe two different modes of topological corner states at each corner and experimentally demonstrate large MCNs without long‐range hopping. Furthermore, based on the HTI, we investigate the influence of various parameters on the phase transition of orbital HOTIs and subsequently construct a phase diagram. Lastly, we theoretically proposed and experimentally demonstrated a general method for constructing TBICs based on orthogonal degenerate *p*‐orbitals. Our work expands the research on ℤ‐classified HOTIs and TBICs to multiple orbitals, which may be beneficial for applications such as acoustic sensing, spatially multimode corner‐emitting topological lasers,^[^ ^52^ ^]^ and non‐Abelian quantum computing.^[^ ^47^, ^53^, ^54^ ^]^

## 7 Experimental Section

### Experimental Setup

All samples used in the experiments were fabricated using photosensitive resin via 3D printing (geometry tolerance of 0.1 mm). This stereolithography material (modulus 3160 MPa, density 1.14 g cm^−3^) was regarded as an acoustic hard boundary for the impedance mismatch. The sound wave was excited by a broadband sound stimulus (Hivi B2S) and the sound pressure amplitude within the sample was measured by a 1/4‐inch‐diameter $Br\ddot{u}el\&Kjær$ type‐4944 microphone. All the data were $\ddot{u}$ processed by the analyzer ($Br\ddot{u}el\&Kjær$ PULSE Type 3160). To facilitate the sound excitation and detection, four holes with the radius of ≈2.5 mm were drilled on the top of each disk‐shaped resonator according to the polarization of the degenerate orthogonal orbitals. These holes should be sealed when not in use.

For generating orbital sound, a pair of speakers connected to a signal generator was positioned as the out‐of‐phase source. In terms of detection, a microphone can be used to measure both the amplitude and phase responses, with another microphone in the same resonator serving as the phase reference, as illustrated by the sketched microphones in Figure 2a. The captured sound signals, recorded and processed by a network analyzer, can then be utilized to map out the response spectra and pressure‐field distributions.

### Statistical Analysis

All numerical simulations were carried out using COMSOL Multiphysics, a commercial finite‐element solver. The resin material for sample fabrication was modeled as acoustically rigid with an airborne sound speed of 344 m s^−1^. An acoustic crystal with 5  ×  5 unit‐cells was used for the simulation, with soft boundary conditions applied at the ends of all outer coupling tubes to ensure the chiral symmetry of the system.

The HTI sample used in the experiment has dimensions of 612 mm × 609 mm × 17 mm. Several frequency sweep measurements were conducted at the points corresponding to the bulk, edge, and corner (Figure 3a) using both in‐phase and out‐of‐phase dipole sources, respectively, and selected the data that best reflecting the expected topology. Additionally, frequency response optimization by placing the microphone in the anechoic chamber was performed to measure the frequency response curve, which was then used to process the experimental data and mitigate the influence of the microphone's varying response across different frequency ranges on the analysis. Finally, when plotting the sound intensity map, the measured data were normalized.

## Conflict of Interest

The authors declare no conflict of interest.

## Supporting information



Supporting Information

## Data Availability

The data that support the findings of this study are available from the corresponding author upon reasonable request.
