# Supplementary material for: ℤ‐Classified Topological Phases and Bound States in the Continuum Induced by Multiple Orbitals
Source: Adv Sci (Weinh). 2025 Jan 21;12(10):2409574. doi: 10.1002/advs.202409574 (PMC11905062; doi:10.1002/advs.202409574)
Supplement: Supplementary file 1 — Supporting Information [file ADVS-12-2409574-s001.docx]

Supplementary Materials for

$\mathbb{Z}$**-C****lassified Topological Phases and** **Bound States in the Continuum Induced by Multiple Orbitals**

Shi-Feng Li^1*^, Wen-Jie Yang^1*^, Cui-Yu-Yang Zhou^1^, Yi-Fan Zhu^2^, Xin-Ye Zou^1†^ and Jian-Chun Cheng^1†^, Badreddine Assouar^3†^

*^1^Key Laboratory of Modern Acoustics, MOE, Institute of Acoustics, Department of*

*Physics, Collaborative Innovation Center of Advanced Microstructures, Nanjing*

*University, Nanjing 210093, People’s Republic of China*

^2^*Jiangsu Key Laboratory for Design and Manufacture of Micro-Nano Biomedical Instruments, School of Mechanical Engineering, Southeast University, Nanjing 211189, China*

^3^*Université de Lorraine, CNRS, Institut Jean Lamour, Nancy, 54000, France.*

^*^These authors contributed equally to this work.

^†^Corresponding authors. Email: [xyzou@nju.edu.cn](mailto:xyzou@nju.edu.cn); [jccheng@nju.edu.cn](mailto:jccheng@nju.edu.cn); badreddine.assouar@univ-lorraine.fr

This supplementary information contains the following sections:

1. Hamiltonian of acoustic orbital 2D SSH crystal.
2. Single-orbital 2D SSH model.
3. Non-integer MCNs.
4. Results of adjusting $u$ in the x direction.
5. Experimental details.
6. TBICs in BKL.
7. The momentum-space Hamiltonian of a hybrid topological insulator (HTI)..

**A. Hamiltonian of acoustic** **orbital 2D SSH crystal.**

The unit-cell of the orbital 2D SSH crystal is depicted in Fig. S1, containing four sites forming a rhombus. One can define three unit-vectors along the hopping directions between the nearest-neighboring sites as shown in Fig. S1.

|  | $\boldsymbol{e}_{\boldsymbol{1}}=\left( 1,0 \right);\boldsymbol{e}_{\boldsymbol{2}}=\left( -sin\theta,cos\theta\right);\boldsymbol{e}_{\boldsymbol{3}}=\left( -sin\theta,-cos\theta\right)$ | (1) |
| --- | --- | --- |

Now we consider that one lattice site can allows for two orbitals, i.e., the $p_{x}$ and $p_{y}$ orbitals. Due to the spatial orientation of the $p$*-*orbitals, there are two types of hopping processes between the $p$*-*orbitals, which produce the $\sigma$ and $\pi$ types of bonds, respectively. The $\sigma$-type hopping $t_{\sigma}$ describes the hopping on neighboring sites with the orientation of $p$*-*orbitals along the bond direction, while the $\pi$-type hopping. $t_{\pi}$ describes the hopping with orientation perpendicular to the bond direction, as shown in Figure 1(a). In the approximation of tight binding, the annihilate operator for the *p-*orbital can be defined on the $p_{x}$ and $p_{y}$ basis: $\boldsymbol{p} = {( p_{x}, p_{y} )}^{T}$. For the $\sigma$- bonding, the *p* orbital should be projected along the hopping directions of the lattice, i.e., the

|  | $p_{1}=\boldsymbol{p\cdot}\boldsymbol{e}_{\boldsymbol{1}}=\left( p_{x},0 \right);$  $p_{2,3}=\boldsymbol{p\cdot}\boldsymbol{e}_{\boldsymbol{2,3}}\boldsymbol{=}\left( -sin\theta* p_{x},\pm cos\theta* p_{y} \right);$ | (2) |
| --- | --- | --- |

Similarly, for the $\pi$- bonding, the $p$*-*orbital should be projected along directions $\boldsymbol{d}$ perpendicular to $\boldsymbol{e}$, with the projected operators being

|  | ${p_{1}}^{'}=\boldsymbol{p\cdot}\boldsymbol{d}_{\boldsymbol{1}}=\left( 0, p_{y} \right);$  $p_{2,3}=\boldsymbol{p\cdot}\boldsymbol{d}_{\boldsymbol{2,3}}\boldsymbol{=}(\mp cos\theta* p_{x},-sin\theta* p_{y} );$ | (3) |
| --- | --- | --- |

The real-space Hamiltonian *p*- orbital 2D SSH crystal reads

|  | $H=\sum_{\boldsymbol{r}} {[t}_{1\sigma}(a_{\boldsymbol{r},1}{b_{\boldsymbol{r},1}}^{\dagger}+b_{\boldsymbol{r},2}{c_{\boldsymbol{r},2}}^{\dagger}+c_{\boldsymbol{r},1}{d_{\boldsymbol{r},1}}^{\dagger}+d_{\boldsymbol{r},2}{a_{\boldsymbol{r},2}}^{\dagger})$  +$t_{1\pi}({a_{\boldsymbol{r},1}}^{'}{b_{\boldsymbol{r},1}}^{'\dagger}+{b_{\boldsymbol{r},2}}^{'}{c_{\boldsymbol{r},2}}^{'\dagger}+{c_{\boldsymbol{r},1}}^{'}{d_{\boldsymbol{r},1}}^{'\dagger}+{d_{\boldsymbol{r},3}}^{'}{a_{\boldsymbol{r},3}}^{'\dagger})$  +$t_{2\sigma}(b_{\boldsymbol{r-}\boldsymbol{e}_{\boldsymbol{1}},1}{a_{\boldsymbol{r-}\boldsymbol{e}_{\boldsymbol{1}},1}}^{\dagger}+c_{\boldsymbol{r-}\boldsymbol{e}_{\boldsymbol{1}},2}{d_{\boldsymbol{r},2}}^{\dagger}+d_{\boldsymbol{r-}\boldsymbol{e}_{\boldsymbol{2}},1}{a_{\boldsymbol{r-}\boldsymbol{e}_{\boldsymbol{2}},1}}^{\dagger}+c_{\boldsymbol{r-}\boldsymbol{e}_{\boldsymbol{2}},3}{b_{\boldsymbol{r-}\boldsymbol{e}_{\boldsymbol{2}},3}}^{\dagger})$  +$t_{2\pi}({b_{\boldsymbol{r-}\boldsymbol{e}_{\boldsymbol{1}},1}}^{'}{a_{\boldsymbol{r-}\boldsymbol{e}_{\boldsymbol{1}},1}}^{'\dagger}+{c_{\boldsymbol{r-}\boldsymbol{e}_{\boldsymbol{1}},3}}^{'}{d_{\boldsymbol{r},3}}^{'\dagger}+{d_{\boldsymbol{r-}\boldsymbol{e}_{\boldsymbol{2}},1}}^{'}{a_{\boldsymbol{r-}\boldsymbol{e}_{\boldsymbol{2}},1}}^{'\dagger}+{c_{\boldsymbol{r-}\boldsymbol{e}_{\boldsymbol{2}},3}}^{'}{b_{\boldsymbol{r-}\boldsymbol{e}_{\boldsymbol{2}},3}}^{'\dagger})]+h.c.$ | (4) |
| --- | --- | --- |

Here, $a_{\boldsymbol{r}}/{a_{\boldsymbol{r}}}^{'}$, $b_{\boldsymbol{r}}/{b_{\boldsymbol{r}}}^{'}$, $c_{\boldsymbol{r}}/{c_{\boldsymbol{r}}}^{'}$ and $d_{\boldsymbol{r}}/{d_{\boldsymbol{r}}}^{'}$, are the $\sigma/\pi$ types of projection operators associated with three sites A, B, and C in a unit-cell, respectively, located at position **r**. The subscript$i$ in operator $a_{\boldsymbol{r},i}$ denotes the projection direction along $\boldsymbol{e}_{i}$ with $i = 1, 2, 3$. $t_{1\sigma}$ and $t_{1\pi}$ ($t_{2\sigma}$ and $t_{2\pi}$) are the intra- (inter-) cell $\sigma$ and $\pi$ types of hopping amplitude, which are determined by the width of the intra- (inter-) coupling tubes $d_{x1},d_{y1} (d_{x2},d_{y2})$ shown Figure 1(c). The Hamiltonian demonstrates that the acoustic crystal still respects chiral symmetry after the introduction of ODoF.


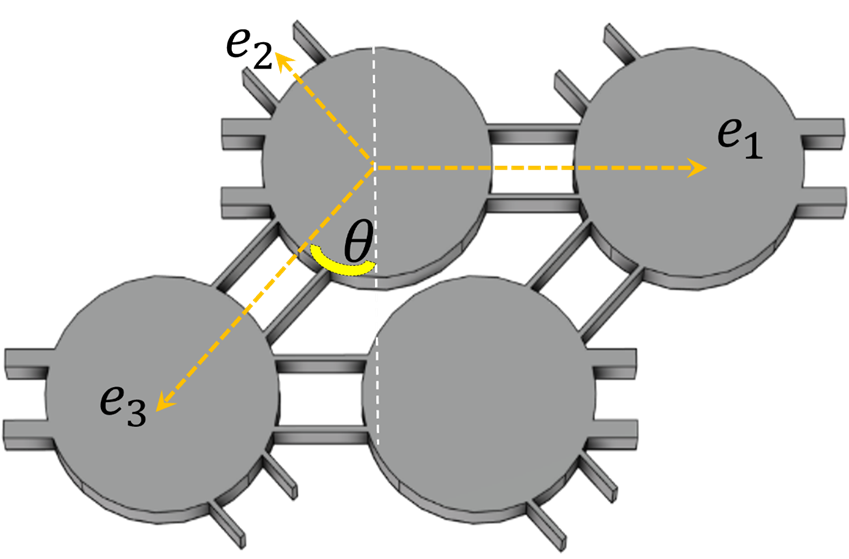


FIG. S1. Schematic diagram of unit-cell of the acoustic orbital 2D SSH lattice. $\boldsymbol{e}_{1}$, $\boldsymbol{e}_{2}$, and $\boldsymbol{e}_{3}$ are three hopping directions.

**B. Single-orbital 2D SSH model.**

Generally, the boundary-obstructed topological phase induced by the charge fractionalization are at the mid-gap when particle-hole symmetry or chiral symmetry is preserved, and the observation of these states always requires spectral isolation. However, the lattices with $C_{4v}$ symmetry as well as chiral symmetry, the degenerate bands at the high symmetry points, always result in the gap being closed, which naturally hinders the identification of the topological zero-energy states. For a conventional single-orbital 2D SSH model, keeping the intra- and inter-cell hopping amplitude in the y direction as $t_{1y}=1$, $t_{2y}=5$, respectively, as well as keeping the intra-cell hopping in the x direction as $t_{1x}=1$, and changing the intercell hopping amplitude $t_{2x}$, the eigenvalues spectrum is shown in Fig. S2(b). It can be seen that when $t_{2x}$ is close to$5$ marked by the blue dash line, that is, when the system has $C_{4v}$ symmetry, the bulk bands close and cover the topological corner states marked by the red lines. Fig. S2(b) shows the numerically calculated eigenvalues of a single-orbital 2D SSH acoustic crystal, where the red dots mark the topological corner states. However, they are mixed in with the bulk/edge states also at zero energy, marked by black dots. The two insets show the sound field distributions corresponding to one of the red dots (topological corner state) and one of the black dots (bulk/edge state), from which the mixing of corner states and bulk states can be clearly seen.


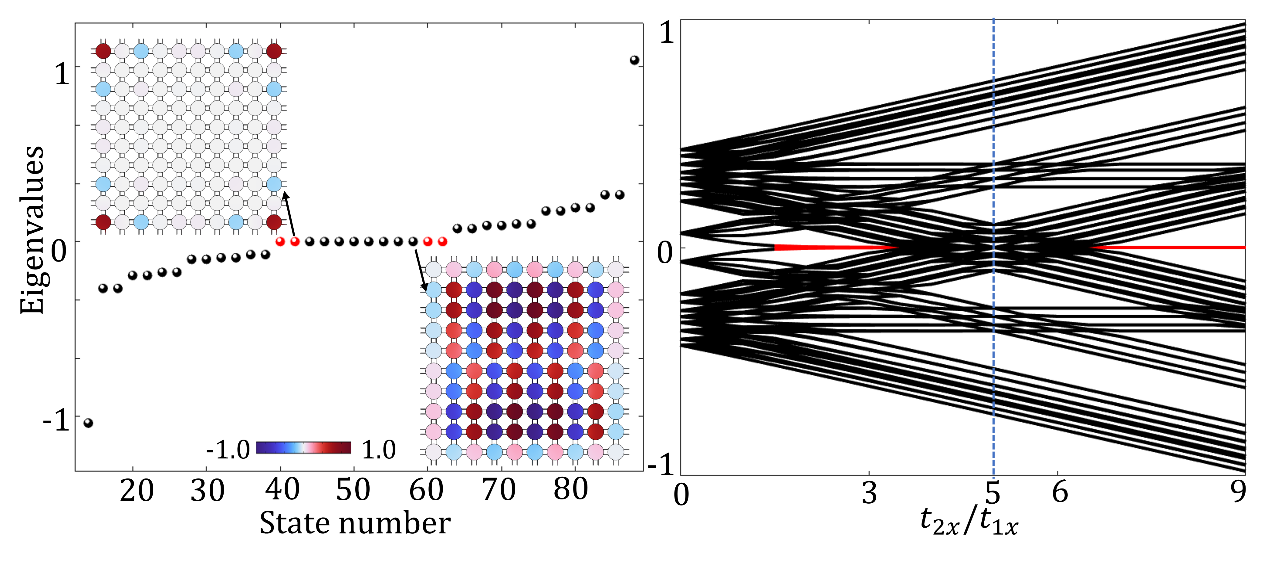


FIG. S2. Eigenvalues and eigenvalue spectrum of the conventional single-orbital 2D SSH model. In the left panel, black dots indicate bulk/edge states, while red dots mark the topological corner states. The inset illustrates the acoustic field distribution at one red dot and one black dot. In the right panel, red lines denote zero-energy modes.

**C. Non-integer MCNs.**

In fact, the MCNs are topological invariants that generalize the classification provided by the 1D winding number to higher-dimensional systems i.e., BOTIs. Thus, similar to how the winding number can only remain integer in 1D topological insulators (TIs), integer MCNs only appear in HOTIs, indicating that for gap-closing metallic phases, MCNs may lose their quantized properties. As shown in Fig. S3(a), in the range of $\frac{d_{y2}}{d_{y1}}\in(1.56, 3.12)$, the MCNs of orbital 2D SSH crystal with $\theta= 67.5^{\circ}$ change into decimals between $-1$ and $-2$ with continuous fluctuation, and correspondingly, in the eigenfrequencies spectrum shown in Fig. S3(b), the bulk bands close and the band gap disappears in this range, indicating that the system is in the metallic phase. It should be pointed out that this metallic phase is caused by the isotropy of the system and does not affect the existence and distribution of the topological corner states.


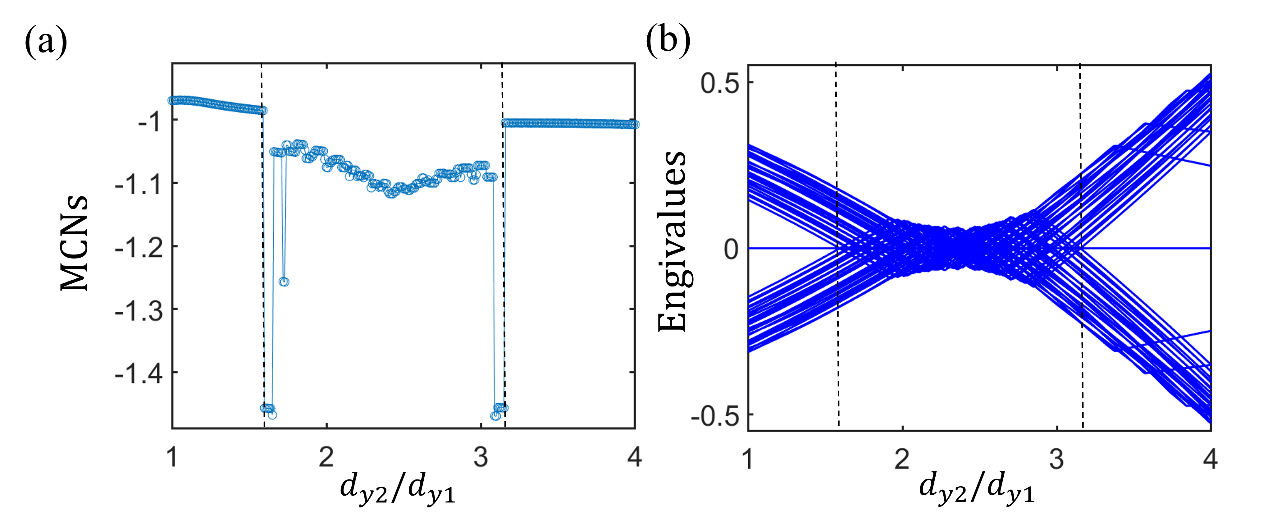


FIG. S3. MCNs and band structure of the orbital 2D SSH model with a fixed θ=67.5° with respect to $\frac{d_{y2}}{d_{y1}}$. (a) Within a certain range $\frac{d_{y2}}{d_{y1}}\in(1.56, 3.12)$, the MCNs exhibit non-integer values and fluctuate continuously. (b) Within the range $\frac{d_{y2}}{d_{y1}}\in(1.56, 3.12)$, the bulk bands close, resulting in the disappearance of the band gap.

**D. Results of adjusting** $\boldsymbol{u}$ **in the x direction.**

In the manuscript, we have shown that, tuning the distance $u$ between the waveguides in the y direction can induce topological corner states in an otherwise topologically trivial TJI. Here, we show that tuning the distance between the waveguides in the x direction can also lead to the similar effect. As shown in Fig. S4(a), for a TJI with the inter-cell coupling tubes in the y direction having width $d_{2}$, while other tubes have width $d_{1}$, ($d_{2}=4*d_{1}$), and at the same time, the distance between the intra-cell coupling tubes in the x direction $u$ is varied from $0$ to $u_{0}$, and the corresponding eigenfrequency spectrum is shown in Fig. S4(c). The red line marking the zero-energy modes emerge as long as $u/ u_{0}\leq0.65$. As shown in Fig. S4(b), the corner states marked by red dots exhibit $p_{y}$ modes that demonstrate their correspondence to zero-energy modes of the BBH model.


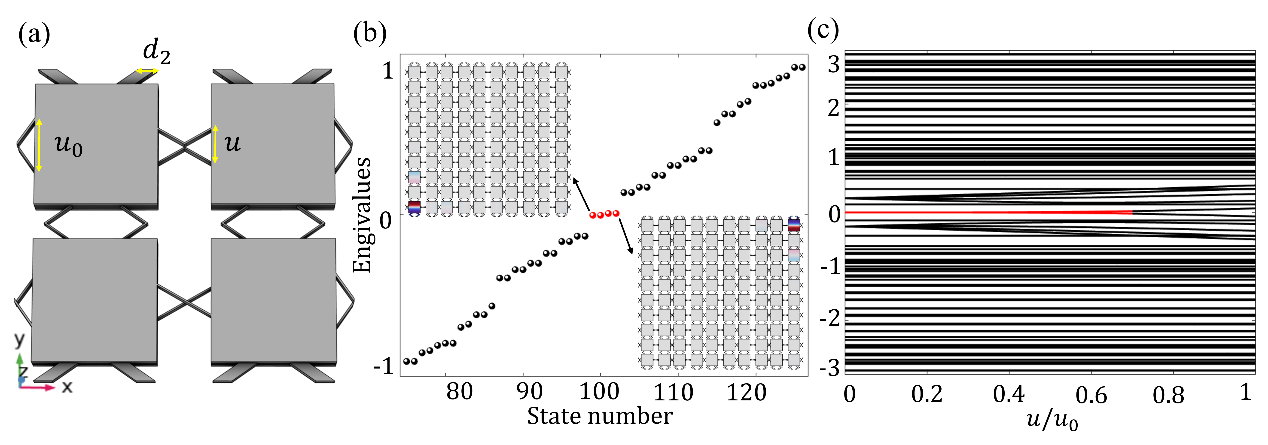


FIG. S4. (a) Schematic diagram of unit-cell of the acoustic orbital TJI lattice, with varing distance $u$ of the inter-cell coupling tubes in the x direction. (b) Eigenvalues of the TJI at $\frac{u}{u_{0}}=0.45$, where red and black dots denote topological corner states and bulk/edge states, respectively. The insets show the acoustic field distribution of the topological corner states, demonstrating the y-polarization of the corner states. (c) Eigenvalue spectrum of the TJI, with the red line marking the zero-energy mode.

**E. Experimental details.**

All samples used in the experiments are fabricated using photosensitive resin via 3D printing (geometry tolerance of $0.1 mm$). This stereolithography material (modulus $3160 MPa$, density $1.14 g/{cm}^{3}$) is regarded as an acoustic hard boundary for the impedance mismatch. The sound wave is excited by a broadband sound stimulus (Hivi B2S), and the sound pressure amplitude within the sample is measured by a 1/4-inch-diameter $Br\ddot{u}el\&Kjær$ type-4944 microphone. All the data are processed by the analyzer ($Br\ddot{u}el\&Kjær$ PULSE Type 3160). To facilitate the sound excitation and detection, four holes with the radius of ~2.5 mm were drilled on the top of each disk-shaped resonator according to the polarization of the degenerate orthogonal orbitals. These holes should be sealed when not in use.

For generating orbital sound, a pair of speakers connected to a signal generator is positioned as the out-of-phase source. In terms of detection, a microphone can be used to measure both the amplitude and phase responses, with another microphone in the same resonator serving as the phase reference, as illustrated by the sketched microphones in Figure 2(a). The captured sound signals, recorded and processed by a network analyzer, can then be utilized to map out the response spectra and pressure-field distributions.

**F. TBICs in BKL.**

In order to conveniently demonstrate that the TBICs proposed in the text are the result of the distinct influences of two orthogonal polarization modes on the onsite energy, we have chosen the one-dimensional SSH model where the $p_{x}$ and $p_{y}$ modes are completely decoupled as an example in the manuscript. Indeed, as long as the difference between σ and π bonds can lead to different offsets of the zero-energy mode, it can be used to construct TBICs. This does not even require protection by chiral symmetry, such as the BKL model with generalized chiral symmetry. In the acoustic orbital BKL crystal shown in Fig. S5(a), we can control the strength of $\pi$-bonding by adjusting the spacing between the coupled tubes. Fig. S5(b) and Fig. S5(d) respectively show the eigenvalue spectra of the numerical and TBM theory calculations regarding $t_{\pi}/t_{\sigma}$, on both of which the red lines indicate zero-energy states that cross the bulk/edge bands marked by black lines, demonstrating the existence of TBICs.

For an orbital BKL lattice with $\frac{t_{\pi}}{t_{\sigma}}=0.4$ , we numerically calculate the sound field excited by a dipole sound source with a frequency of 3.92 kHz placed in the second cavity in the lower left corner of the sample. As shown in Fig. S5(c), for the $p_{x}$ mode sound source, the sound energy is almost completely localized in the lower left corner cavity, while for the $p_{y}$ mode source, the sound field distribution shows obvious edge states. This orbital-selective sound field distribution undoubtedly demonstrates the existence of TBICs in the orbital BKL.


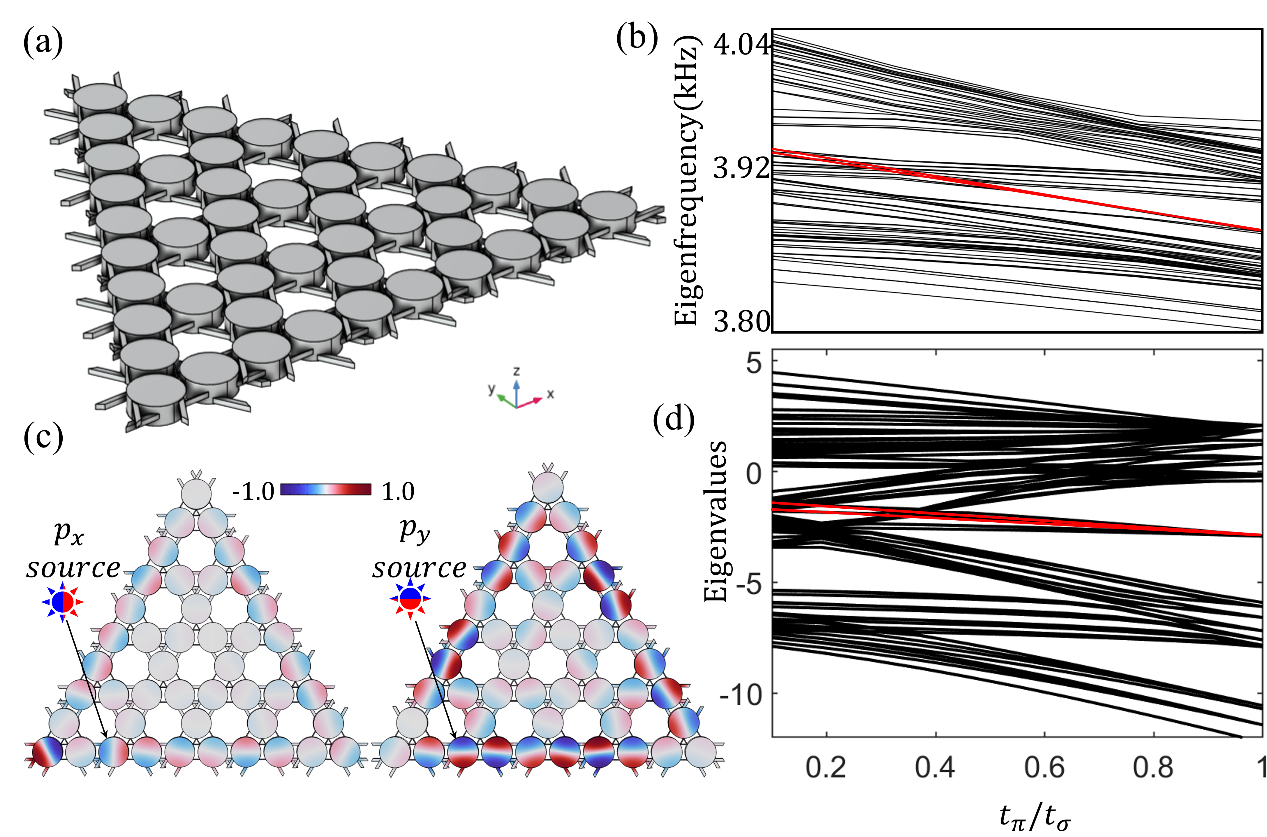


FIG. S5. (a) Schematic diagram of the acoustic orbital BKL lattice. (b) The numerically calculated eigenvalue spectrum with regarding to $t_{\pi}/t_{\sigma}$, and the red lines mark the zero-energy states. (c) The sound fields excited by a $p_{x}$ and $p_{y}$ sound source with a frequency of 3.92 kHz, respectively. (d) The eigenvalue spectrum of the orbital BKL lattice calculated by TBM theory.

**G. The momentum-space Hamiltonian of a** **hybrid topological insulator (HTI).**

Here, we present the momentum-space Hamiltonian of the HTI and use a unitary transformation to decouple it into a separate 2D SSH model and a topological quadrupole insulator. By performing a Fourier transform on Eq. (2) in the manuscript and introducing an eight-component spinor$\psi=\left[ a_{\mathbf{k},x},a_{\mathbf{k},y},b_{\mathbf{k},x},b_{\mathbf{k},y},c_{\mathbf{k},x},c_{\mathbf{k},y},d_{\mathbf{k},x},d_{\mathbf{k},y}, \right]^{T}$, the Hamiltonian can be written into $H=\sum_{\mathbf{k}} \psi^{\dagger}H(\mathbf{k})\psi$. The matrix $H(\boldsymbol{k})$takes the structure as

|  | $H=\left[ \begin{matrix} 0 & D_{1} & 0 & D_{2} \\ {D_{1}}^{\dagger} & 0 & D_{2} & 0 \\ 0 & {D_{2}}^{\dagger} & 0 & D_{3} \\ {D_{2}}^{\dagger} & 0 & {D_{3}}^{\dagger} & 0 \end{matrix} \right]$, | (5) |
| --- | --- | --- |

in which the 2*2 matrices $D_{1}$, $D_{2}$ and $D_{3}$ are

|  | $D_{1}=\left[ \begin{matrix} t_{1\sigma}+t_{2\sigma}*e^{-ik_{x}} & 0 \\ 0 & t_{1\pi}+t_{2\pi}*e^{-ik_{x}} \end{matrix} \right]$, | (6) |
| --- | --- | --- |
|  | $D_{2}=\left[ \begin{matrix} t_{1\pi}+t_{2\pi}*e^{-ik_{y}} & 0 \\ 0 & t_{1\sigma}+t_{2\sigma}*e^{-ik_{y}} \end{matrix} \right]$, | (7) |
|  | $D_{3}=\left[ \begin{matrix} t_{1\sigma}+t_{2\sigma}*e^{-ik_{x}} & 0 \\ 0 & {-t}_{1\pi}-t_{2\pi}*e^{-ik_{x}} \end{matrix} \right].$ | (8) |

By applying a unitary transformation as follow, $H(\boldsymbol{k})$can be transformed into a new matrix $H_{decouple}(\boldsymbol{k})$, which consists of two decoupled components.

|  | $H_{decouple}\left( \boldsymbol{k} \right)=UH(\boldsymbol{k})U^{T}=\left[ \begin{matrix} H_{2D-SSH} & 0 \\ 0 & H_{quadrapole} \end{matrix} \right]$, | (9) |
| --- | --- | --- |

where the unitary matrix is $U=\left[ \begin{matrix} 1 & 0 & 0 & 0 & 0 & 0 & 0 & 0 \\ 0 & 0 & 1 & 0 & 0 & 0 & 0 & 0 \\ 0 & 0 & 0 & 0 & 1 & 0 & 0 & 0 \\ 0 & 0 & 0 & 0 & 0 & 0 & 1 & 0 \\ 0 & 1 & 0 & 0 & 0 & 0 & 0 & 0 \\ 0 & 0 & 0 & 1 & 0 & 0 & 0 & 0 \\ 0 & 0 & 0 & 0 & 0 & 1 & 0 & 0 \\ 0 & 0 & 0 & 0 & 0 & 0 & 0 & 1 \end{matrix} \right]$, and the decouple sub- Hamiltonians are

$H_{2D-SSH}=\left[ \begin{matrix} 0 & t_{1\sigma}+t_{2\sigma}*e^{-ik_{x}} & 0 & t_{1\pi}+t_{2\pi}*e^{-ik_{y}} \\ t_{1\sigma}+t_{2\sigma}*e^{ik_{x}} & 0 & t_{1\pi}+t_{2\pi}*e^{-ik_{y}} & 0 \\ 0 & t_{1\pi}+t_{2\pi}*e^{ik_{y}} & 0 & t_{1\sigma}+t_{2\sigma}*e^{-ik_{x}} \\ t_{1\pi}+t_{2\pi}*e^{ik_{y}} & 0 & t_{1\sigma}+t_{2\sigma}*e^{ik_{x}} & 0 \end{matrix} \right]$, $H_{quadrapole}=\left[ \begin{matrix} 0 & t_{1\pi}+t_{2\pi}*e^{-ik_{x}} & 0 & t_{1\sigma}+t_{2\sigma}*e^{-ik_{y}} \\ t_{1\pi}+t_{2\pi}*e^{ik_{x}} & 0 & t_{1\sigma}+t_{2\sigma}*e^{-ik_{y}} & 0 \\ 0 & t_{1\sigma}+t_{2\sigma}*e^{ik_{y}} & 0 & {-t}_{1\pi}-t_{2\pi}*e^{-ik_{x}} \\ t_{1\sigma}+t_{2\sigma}*e^{ik_{y}} & 0 & {-t}_{1\pi}-t_{2\pi}*e^{ik_{x}} & 0 \end{matrix} \right]$, respectively. The feasibility of such decoupling arises from the orthogonality of the $p_{y}$ and $p_{x}$ orbitals, with the two orbitals corresponding to two separate sub-Hamiltonians.
